# A randomized, double-blind, placebo-controlled phase I trial of inhalation treatment of recombinant TFF2-IFN protein: A multifunctional candidate for the treatment of COVID-19

**DOI:** 10.3389/fphar.2022.1063106

**Published:** 2022-12-12

**Authors:** Yan Liu, Guanxing Zhai, Weihui Fu, Xiaoyan Zhang, Jianqing Xu

**Affiliations:** ^1^ Institutes of Biomedical Sciences, Shanghai Medical College, Fudan University, Shanghai, China; ^2^ National Research Center for Translational Medicine at Shanghai, Ruijin Hospital Affiliated to Shanghai Jiao Tong University School of Medicine, Shanghai, China; ^3^ Shanghai Public Health Clinical Center, Fudan University, Shanghai, China

**Keywords:** respiratory viral infection, COVID-19, IFN, TFF2, safety

## Abstract

**Background and Objectives:** Coronavirus disease 2019 (COVID-19) has caused global pandemics in the last 3 years, and the development of new therapeutics is urgently needed. This study aimed to assess the safety, tolerated, and prolonged retention of recombinant protein trefoil factor 2 (TFF2)- interferon (IFN) in the respiratory tract of healthy volunteers.

**Methods:** We conducted a randomized, double-blind, placebo-controlled, single-dose, dose-escalation phase I study to evaluate safety, tolerability, pharmacokinetics (PK), and cytokine responses after administration of recombinant TFF2-IFN proteins. Healthy volunteers were informed, enrolled, and randomized into four groups with a dose escalation of 0.2, 1, 2, and 4 mg and then inhaled the investigation product or placebo. Thirty-two eligible participants were finally enrolled; eight were assigned to the placebo group and 24 to the TFF2-IFN group, with six participants per group. Data were collected from 19 November 2021, to 4 January 2022.

**Results:** All 32 participants completed the study. Of the participants who received the recombinant TFF2-IFN protein, 41.7% (10/24) reported 11 adverse events (AEs) during treatment and 62.5% (5/8) of those who received a placebo reported six AEs. Sixteen of the 17 AEs were grade 1. Only one grade 3 AE occurred in the placebo group and no worse event occurred as a serious adverse event. The pharmacokinetics was analyzed for times and concentrations of the investigation products in 0.2, 1, 2, and 4 mg groups in 24 recipients of TFF2-IFN, and the results showed that TFF2-IFN was retained in the lung for at least 6–8 h. Only the highest dose group (4 mg) had a transient detectable concentration in serum, while all other dose groups had a level below the lower limit of quantification.

**Conclusion:** In this study, the recombinant TFF2-IFN protein was a well-tolerated and safe therapeutic when administered by nebulization, characterized by prolonged retention in the respiratory tract, which would be greatly beneficial in combating respiratory viral infection.

**Systematic Review Registration**: [http://www.chictr.org.cn], identifier [ChiCTR2000035633].

## Introduction

To date, 539 million confirmed cases of coronavirus disease 2019 (COVID-19) have been reported, with more than 6 million deaths reported to the WHO. Severe acute respiratory syndrome coronavirus-2 (SARS-CoV-2) is highly contagious and can cause life-threatening viral pneumonia. On 16 May 2022, a total of 12 billion vaccine doses had been administered, and morbidity and mortality in COVID-19 had been greatly reduced. However, the monthly incidence of breakthrough infections increased from July to November 2021 in both cohorts, with a higher incidence in the BNT162b2 cohort than in the mRNA-1273 cohort, and reached 2.8 and 1.6 cases per 1000 person-days in November 2021, respectively (Wang et al., 2022), indicating that even people vaccinated with effective COVID-19 vaccines remain vulnerable to SARS-CoV-2. Therefore, safe and effective drugs are urgently needed to minimize hospitalization rates and prevent the development of severe diseases.

Intravenously administered remdesivir (NIH. COVID-19 Treatment Guideline Panel, 2020), neutralizing antibodies (DeFrancesco, 2020) or orally administered molnupiravir (Pruijssers and Denison, 2019) and Paxlovid (Mahase and Kmietowicz, 2021; Wen et al., 2022) have been proven to be effective in the prevention of severe infections. The primary sites of SARS-CoV-2 infection are the respiratory tract and lungs, and systemic administration of drugs is delayed and diluted to reach the infection sites, thus crippling its effectiveness in containing viral replication. In contrast, inhaled medicine primarily concentrates on the respiratory tract and therefore takes a stronger and earlier action than systemic administration. In addition, this route is likely to reduce the exposure of systemic organs and subsequently minimize systemic side effects (Mason, 2020). Overall, the development of novel, safe and effective inhaled therapeutics for COVID-19, influenza, and other respiratory viral infections has remained an unmet clinical need.

Effective antiviral responses require the sequential engagement of innate and adaptive immune responses. Innate responses driven by viruses and their derivatives are usually characterized by the induction of interferon (IFN) production. Infected cells release IFN to activate the antiviral IFN-stimulating genes (ISGs) of nearby cells and induce the production of inflammatory cytokines (Walz et al., 2021). The antiviral action of IFNs involves several mechanisms. For example, protein kinase R is elicited by IFNs signaling to trigger autophosphorylation upon viral infection (Toth et al., 2006). Interestingly, IFNs also activate 2’−5’ oligoadenylate synthesis (OAS) to phosphorylate targeted proteins (Sadler and Williams, 2008). In addition, IFNs activate nitric oxide synthase and several other pathways (Samuel, 2001; Lamers et al., 2019). In fact, in the early stages of viral infection, IFNs exert a high antiviral activity by provoking natural killer cells and dendritic cells (Arico et al., 2020). Type I IFN protects against SARS-CoV-2 infection (King and Sprent, 2021).

Studies have shown that type I IFNs play a dual role in SARS-CoV-2-induced responses. First, it could provide a highly protective effect before the viral peak and inflammatory phase of the disease. In contrast, type I IFN may cause long‐lasting harm and immunopathology in patients when they appear or are implemented during the inflammatory and severe stages of the disease (Sodeifian et al., 2022). Therefore, treatment in the early stages of COVID-19 with type I IFN is likely to slow disease progression by containing the replication of SARS-CoV-2 in the host (Masood et al., 2021).

Trefoil factor 2 (TFF2) is a secreted polypeptide, that is, resistant to acid, heat, and protease hydrolysis and can protect the gastrointestinal tract and respiratory tract from microbial injury by promoting the repair of injury and regulating the immune response (Poulsen et al., 1999) (Baus-Loncar et al., 2005) (Farrell et al., 2002). Our experiments in mice with an influenza virus model showed that TFF2 could reduce morbidity and mortality by attenuating inflammatory responses and repairing pulmonary function, but not by viral replication (Patent application No:201610104936.8).

Based on these results, we hypothesized that the combination of TFF2 and human type I IFN is likely to synergistically fight against COVID-19 by reducing viral replication, alleviating the inflammatory response, and improving respiratory mucosal reconstruction. We have previously tested this concept in SARS-CoV-2 infected patients during the first wave of the COVID-19 outbreak in 2020, and the results showed that the combination of TFF2 and human IFN kappa, one of the type I IFNs, is a highly safe and effective therapeutic for COVID-19 (Fu et al., 2020a; Fu et al., 2020b). Subsequently, we optimized the design to construct a recombinant TFF2-IFN protein and showed that this molecule has the dual functions of inhibiting virus replication, regulating the immune response (data not shown), and repairing the respiratory mucosa. Herein, we report the findings of a randomized, double-blind, placebo-controlled phase I trial of inhalation treatment with the recombinant TFF2-IFN protein in healthy volunteers: a multifunctional candidate for the treatment of COVID-19, in preparation for therapeutic evaluation of COVID-19, influenza, and other respiratory viral infections.

## Materials and methods

### Study design

This was a single-center, randomized, double-blind, placebo-controlled phase I study to assess the safety and PK of the recombinant TFF2-IFN protein in healthy volunteers. The first volunteer was enrolled on 15 November 2021, and the last 7-day visits were completed on 4 January 2022.

The trial was conducted following the principles of the Declaration of Helsinki, Good Clinical Practices, and the International Council for Harmonization E-9 guidelines, as well as all applicable laws and regulations. The protocol and all amendments were reviewed and approved by the Shanghai Public Health Clinical Center’s Ethics Committee. The study was registered with the Chinese Clinical Trial Register (http://www.chictr.org.cn), ChiCTR2000035633, and was conducted at the Shanghai Public Health Clinical Center. The protocol and informed consent were reviewed and approved by the ethics committee. Written informed consent was obtained from all participants. This study is reported following the Consolidated Standards of Reporting Trials (CONSORT) reporting guidelines.

### Participants

Males and non-pregnant and non-lactating females who were abstinent or who agreed to use effective contraceptive methods throughout the course of the study were eligible. Eligible participants were aged ≥18 and ≤60 years and were normally active and in good health with no current chronic diseases and normal physical examination. The participants underwent an electrocardiogram (ECG) without clinically significant abnormalities. None of the participants weighed <50 kg for males and <45 kg for females. Healthy volunteers were excluded if they had a severe clinically significant allergy, current acute or chronic disease, particularly respiratory disease, kidney damage, underlying disease that could interfere with inhalation of the study product, or alcohol consumption within 24 h before administration. Participants were enrolled sequentially according to the dose escalation protocol specified in the protocol.

### Randomization and masking

A randomization list was created using an online research randomizer (https://www.randomizer.org/). The randomization list was created by specifying a 3:1 assignment to “active” or “placebo”. A randomization number was assigned to the investigation product (IP) and placebo groups before each group; the enrolled subjects were numbered according to the screening. The random list was kept in a closed cabinet and/or electronic file accessible only to the designated unblinded researchers. The treatment label was kept in a sealed envelope. All investigators, participants, clinical and study teams, and pharmacists were masked to the investigational protocol.

### Intervention and assessments

In the dose escalation scheme, four doses of recombinant TFF2-IFN (0.2, 1, 2, and 4 mg) were administered *via* aerosol inhalation. The recombinant TFF2-IFN protein was produced under Good Manufacturing Practices conditions, with a purity of more than 95%, and the biological activities of the recombinant protein were verified *in vitro*. Placebo was 0.9% sodium chloride. In addition, the candidate was administered by nasal inhalation. The products were dissolved in 5 ml of sterilized water, and the aerosol was administered for 20 ± 5 min using a nasal mask driven by a medical compressed air atomizer (YUWELL, 403M). Throughout the study, both recombinant TFF2-IFN and placebo were administered by qualified study staff. This randomized, single-center, placebo-controlled, double-blind study was conducted in the 0.2, 1, 2, and 4 mg groups [six assigned to active and two to placebo (*n* = 8)]. Screening and enrollment were performed sequentially for one cohort after the other. A higher dose was administered after the safety assessment of all subjects in the pre-dose group was completed. Each participant received only a single dose. The duration of aerosol inhalation for TFF2-IFN was approximately 20 ± 5 min.

General physical examination, ECG, laboratory tests, and urinalysis were performed before inhalation 2 h, and 48 h after aerosol inhalation. All adverse events (AEs) were recorded during the 10-day period after administration, and serious adverse events (SAEs) were assessed throughout the study. AEs were coded using the Medical Dictionary for Regulatory Activities (MedDRA, 23.0).

Blood samples for pharmacokinetics (PK) assessment of recombinant TFF2-IFN protein were collected at baseline (day 0) and then on day 1 at 0.25, 0.5, 1, 1.5, 2, 2.5, 3, 4, 6, 8, 10, 24, and 48 h after administration. The serum concentrations of TFF2-IFN were determined using an in-house validation enzyme-linked immunosorbent assay (ELISA) method. The linear range of this method was 50.0–800 pg/ml. Blood samples for Cytokines analysis were collected at 0 h (within 1 h before administration), 2, 8, and 24 h after administration. IL-6, IFN-γ, IL-10, TNF-α as biomarkers were analyed using the Simoa CorPlex Human Cytokine Panel one kit (Cat No: 85–0329). The assay was carried out according to the manufacturer’s (Quanterix, Billerica, MA, United States) protocols.

### Outcomes

The primary objective of this study was to assess the safety of inhalation recombination of the TFF2-IFN protein in healthy volunteers up to day 10. Secondary endpoints were PK and cytokine changes. The safety outcomes were as follows: vital signs, clinical laboratory changes, physical results, AE and SAE, and other safety-related observations. Primary endpoints were defined as frequency of AE, changes in all measured safety variables and baseline, and frequency of overrange values in the placebo and investigational drug groups.

### Statistical analysis

The sample size for each dose group in this study was not based on formal power calculations. The sample size was calculated for purposes sufficient to meet the research objectives and assess safety by obtaining the power required to detect AEs at least once but not for statistical inference. Two sets of populations were analyzed. The safety analysis set included data from all enrolled healthy volunteers who had received any number of investigational products. The significance of the differences was tested using the Chi-squared test and one-way ANOVA with Tukey’s multiple comparison test. Statistical analysis was performed using SAS 9.4 software. The PK analysis set included data from healthy volunteers who received active treatment and did not omit data that influenced the PK assessment. PK analysis was performed in healthy volunteers enrolled in at least one quantifiable drug concentration study. There is no imputation of missing data. Pharmacokinetic analysis was performed using Win-Nonlin 8.2 software. To assess dose proportionality using PK data from cohorts 1–4 using a non-linear power model, the accumulation ratio was calculated for Cmax and area under the curve (AUC) for the IP and placebo groups.

## Results

### Clinical Characteristics

**FIGURE 1:**
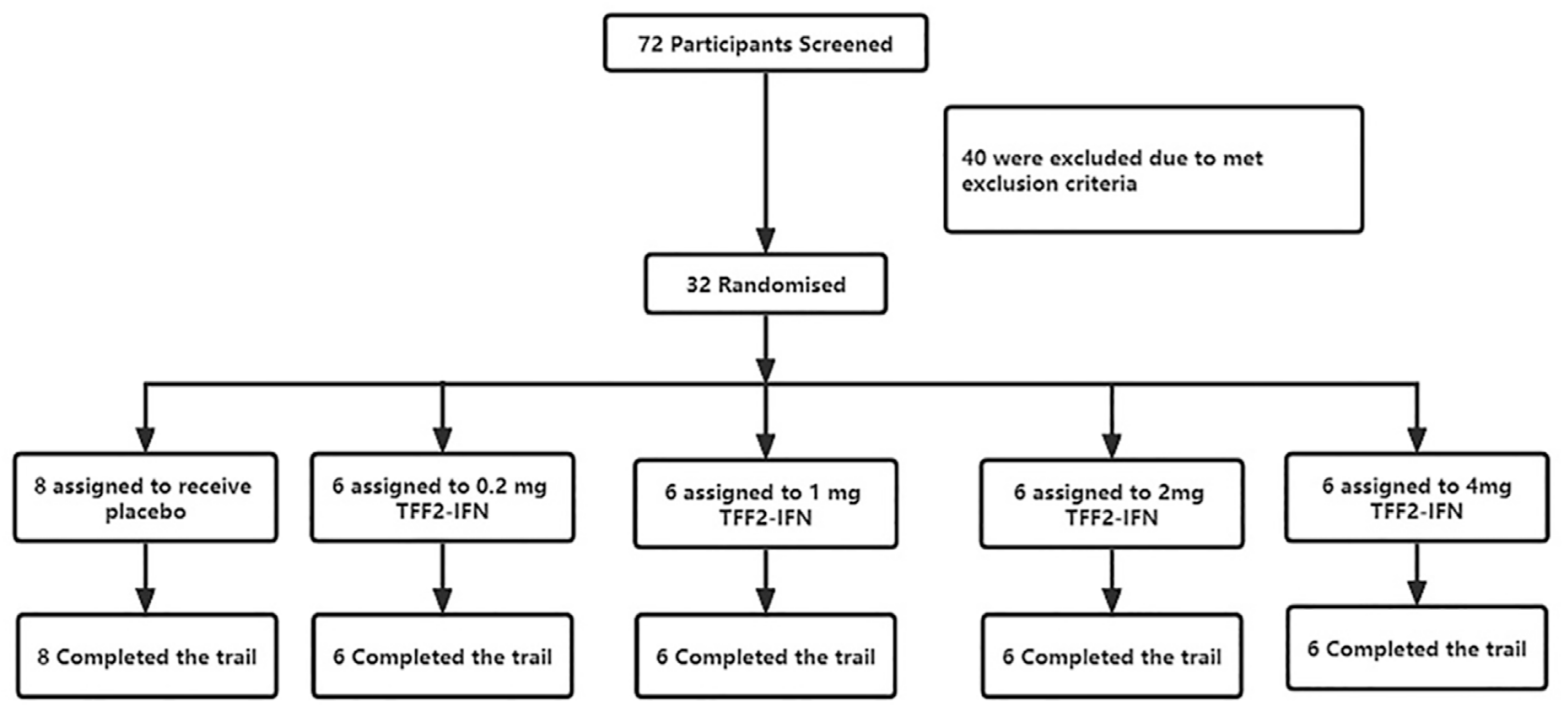
Trial profile.

The study was carried out from 15 November 2021 to 04 January 2022. As shown in the trial flowchart in Figure 1, 72 participants are screened, and 40 are excluded according to the criteria of our approved protocol. Thirty-two participants were finally eligible and enrolled; eight were assigned to the placebo group and 24 to the test group (they received one dose inhalation of the recombinant TFF2-IFN protein. The demographic and clinical characteristics at baseline are summarized in Table 1. There were 15 (46.9%) males and 17 (53.1%) females with a median age of 31.00 years (IQR, 27.00–39.00 years). A median body mass index (BMI) of 22.55 kg/m^2^ (21.30–24.15 kg/m^2^) and median body weight of 61.80 kg (IQR, 57.28–67.50 kg). “Male or female, aged ≥18 years old; BMI in the range of 18–26 kg/m^2^ (inclusion criteria)” was implemented. All 32 subjects completed all examinations and follow-ups as specified in the protocol. For all baseline characteristics, no significant differences were found between the placebo and investigative product groups at each dose.

**TABLE 1:** Demographics and clinical characteristics of the participants in each dose group.

	Test group					Significant
	Placebo (*n* = 8)	0.2 mg (*n* = 6)	1 mg (*n* = 6)	2 mg (*n* = 6)	4 mg (*n* = 6)	*p*-Value
Age (years)						
Median (±IQR)	33.0 (27.50–37.75)	31.0 (28.50–33.75)	34.5 (25.25–47.50)	31.5 (25.25–38.75)	33.0 (26.25–53.00)	ns
Sex						
Male	5 (62.5%)	4 (66.7%)	1 (16.7%)	2 (33.3%)	3 (50%)	ns
Ethnicity						
Han	8 (100%)	6 (100%)	6 (100%)	6 (100%)	6 (100%)	ns
Others	0 (0%)	0 (0%)	0 (0%)	0 (0%)	0 (0%)	
Weight (kg)						
Median (±IQR)	62.85 (58.43–69.68)	64.70 (54.50–70.23)	60.85 (57.30–66.15)	58.95 (55.23–63.48)	62.85 (57.33–67.48)	ns
BMI (kg/m^2^)						
Median (±IQR)	24.00 (20.88–24.98)	21.70 (20.43–23.55)	22.85 (22.00–24.13)	21.75 (20.98–22.90)	22.90 (21.93–23.43)	ns

The significant difference analysis was evaluated using Chi-squared test and one-way ANOVA, with Tukey’s multiple comparison test. (ns: non significant).

### AEs

All 32 participants completed the study and were included in the safety set; 41.7% (10/24) of subjects who received the recombinant TFF2-IFN protein reported 11 AEs during treatment. In contrast, 62.5% (5/8) of the subjects who received a placebo reported six AEs (Table 2). A total of 17 AEs occurred; 16 AEs were grade 1 in terms of severity. Only one grade 3 AE occurred in the placebo group, and no worse AE occurred as SAE. The incidence of AEs in the 0.2, 1, 2, and 4 mg aerosol groups was 16.7%, 16.7%, 50%, and 83.3%, respectively. The incidence of drug-related AEs in the aerosol group was 0%, 0%, 0%, and 50%. The incidence of drug-related AEs was correlated with dose.

**TABLE 2:** Adverse events and their severity and outcome in patients in each dose group.

		Placebo (*n* = 8)		Test group							
				0.2 mg (*n* = 6)		1 mg (*n* = 6)		2 mg (*n* = 6)		4 mg (*n* = 6)	
		No.of AEs	No.of subjects (%)	No.of AEs	No.of subjects (%)	No. Of AEs	No.of subjects (%)	No.of AEs	No.of subjects (%)	No. Of AEs	No.of subjects (%)
Severity	Total	6	5 (62.5%)	1	1 (16.7%)	1	1 (16.7%)	3	3 (50%)	6	5 (83.3%)
	Grade 1	5	4 (50%)	1	1 (16.7%)	1	1 (16.7%)	3	3 (50%)	6	5 (83.3%)
	Grade 2	0	0 (0%)	0	0 (0%)	0	0 (0%)	0	0 (0%)	0	0 (0%)
	Grade 3	1	1 (12.5%)	0	0 (0%)	0	0 (0%)	0	0 (0%)	0	0 (0%)
	Grade 4	0	0 (0%)	0	0 (0%)	0	0 (0%)	0	0 (0%)	0	0 (0%)
	Grade 5	0	0 (0%)	0	0 (0%)	0	0 (0%)	0	0 (0%)	0	0 (0%)
Overcome	Resolved	6 (100%)	—	1 (100%)	—	1 (100%)	—	3 (100%)	—	6 (100%)	—
	Unknown	0	—	0	—	0	—	0	—	0	—

No serious AEs or early discontinuation was reported. The types of AEs in this study included abnormal laboratory test results, infectious diseases, general disorders, and administration site conditions. A total of 17 AEs were observed in each group (Table 3). AEs in TFF2-IFN group or placebo group included infection diseases, nasopharyngitis (16.7% in the TFF2-IFN group and 0% in the placebo group), increased blood glucose (0% in the TFF2-IFN group and 12.5% in the placebo group), and increased creatine phosphokinase (0% in the TFF2-IFN group and 12.5% in the placebo group), neutrophils increased (16.7% in the TFF2-IFN group, 0% in the placebo group), blood chloride increased (16.7% in the TFF2-IFN group, 12.5% in the placebo group), blood uric acid increased (16.7% TFF2-IFN group, 12.5% placebo group), blood creatinine increased (0% in the TFF2-IFN group, 12.5% in the placebo group), platelet count decreased (16.7% in the TFF2-IFN group, 12.5% in the placebo group), increased fibrinogen (16.7% TFF2-IFN group, 0% placebo group), positive occult blood in urine (16.7% on the TFF2-IFN group, 0% in the placebo group), changes in ST wave (16.7% TFF2-IFN group, 12.5% placebo group), sporadic premature beating (16.7% in the TFF2-IFN group, 0% in the placebo group), general disorders and administration site conditions, fever (16.7% in the TFF2-IFN group and 0% in the placebo group).

**TABLE 3:** Incidence of adverse events by system organ classes.

	Placebo (*n* = 8)		0.2 mg (*n* = 6)		1 mg (*n* = 6)		2 mg (*n* = 6)		4 mg (*n* = 6)	
—	No. Of AEs	No. Of subjects (n/N %)	No. Of AEs	No. Of subjects (n/N%)	No. Of AEs	No. Of subjects (n/N%)	No. Of AEs	No. Of subjects (n/N%)	No. Of AEs	No. Of subjects (n/N%)
Total	6	5 (62.5)	1	1 (16.7)	1	1 (16.7)	3	3 (50.0)	6	5 (83.3%)
Infections and infestations										
Nasopharyngitis	0	0 (0%)	0	0 (0%)	0	0 (0%)	1	1 (16.7)	0	0 (0%)
Investigations										
Increased blood glucose	1	1 (12.5%)	0	0 (0%)	0	0 (0%)	0	0 (0%)	0	0 (0%)
Increased blood creatine phosphokinase	1	1 (12.5%)	0	0 (0%)	0	0 (0%)	0	0 (0%)	0	0 (0%)
Increased neutrophil count	0	0 (0%)	0	0 (0%)	0	0 (0%)	0	0 (0%)	1	1 (16.7%)
Increased blood chloride	1	1 (12.5%)	0	0 (0%)	0	0 (0%)	1	1 (16.7%)	0	0 (0%)
Increased Uric Acid	1	1 (12.5%)	0	0 (0%)	0	0 (0%)	1	1 (16.7%)	0	0 (0%)
Increased blood cretinine	1	1 (12.5%)	0	0 (0%)	0	0 (0%)	0	0 (0%)	0	0 (0%)
Decreased platelet count	0	0 (0%)	0	0 (0%)	0	0 (0%)	0	0 (0%)	1	1 (16.7%)
Increased Fibriogen	0	0 (0%)	0	0 (0%)	0	0 (0%)	0	0 (0%)	1	1 (16.7%)
Positive urinary occult blood	0	0 (0%)	0	0 (0%)	0	0 (0%)	0	0 (0%)	1	1 (16.7%)
ST segment abnormal	1	1 (12.5%)	1	1 (16.7%)	1	1 (16.7%)	0	0 (0%)	0	0 (0%)
Occasional premature beat	0	0 (0%)	0	0 (0%)	0	0 (0%)	0	0 (0%)	1	1 (16.7%)
General disorders and administration site conditions										
Pyrexia		0 (0%)		0 (0%)	0	0 (0%)	0	0 (0%)	1	1 (16.7%)

All adverse events are coded using MedDRA, version 22.0.

AEs may related to the TFF2-IFN protein occurred three times in total by the investigator. Nasopharyngitis in 2 mg group. Increased Fibriogen (16.7% in the TFF2-IFN group and 0% in the placebo group), and neutrophils increased (16.7% in the TFF2-IFN group and 0% in the placebo group) and fever (16.7% in the TFF2-IFN group and 0% in the placebo group), which occurred in the highest dose group (4 mg). There were no suspected unexpected serious adverse reactions (SUSAR), discontinuation of the study, or deaths attributed to AEs during the study.

### PK

**FIGURE 2:**
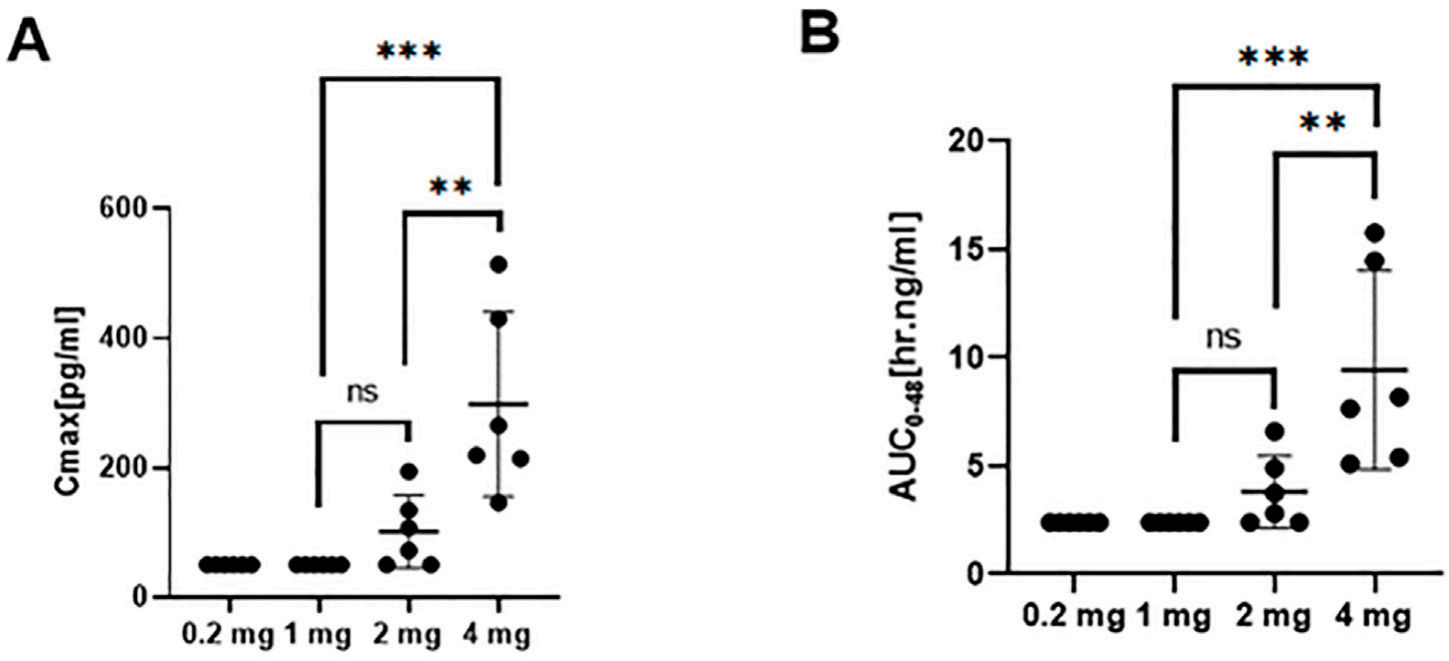
Pharmacokinetic profile (mean ± SD) of TFF2-IFN in plasma in each group. **(A)** Cmax in plasma of each group. **(B)** AUC of each group. Abbreviations: Cmax, maximum concentration; AUC, area under the curve. The significant difference analysis was evaluated using one-way ANOVA with Tukey’s multiple comparison test (*p < 0.05, **p < 0.01, ***p < 0.001, ns *p* > 0.05 non significant).

The PK in 24 subjects was analyzed, including six subjects in each 0.2, 1, 2, and 4 mg groups. The linear range of the recombinant TFF2-IFN protein in sera samples was 50–800 pg/ml, and participants in the 0.2 and 1 mg dose groups had serum levels below the lower limit of quantification. After increasing the inhalation dose to 2 mg, several subjects had a detectable TFF2-IFN concentration in serum samples with an 8 h delay after inhalation. The concentration of TFF2-IFN increased with a 6 h delay in the 4 mg group. The mean C_max_ and AUC_0–48h_ of the 2 mg group were 107 pg/ml and 2.83 ng/ml, and the mean C_max_ and AUC_0–48h_ of the 4 mg group were 306 pg/ml and 14.48 ng/ml, respectively. The times and concentrations detected in the 0.2, 1, 2, and 4 mg groups showed that TFF2-IFN was retained in the lung for at least 6–8 h and then only slightly released into the blood (Figure 2).

**FIGURE 3:**
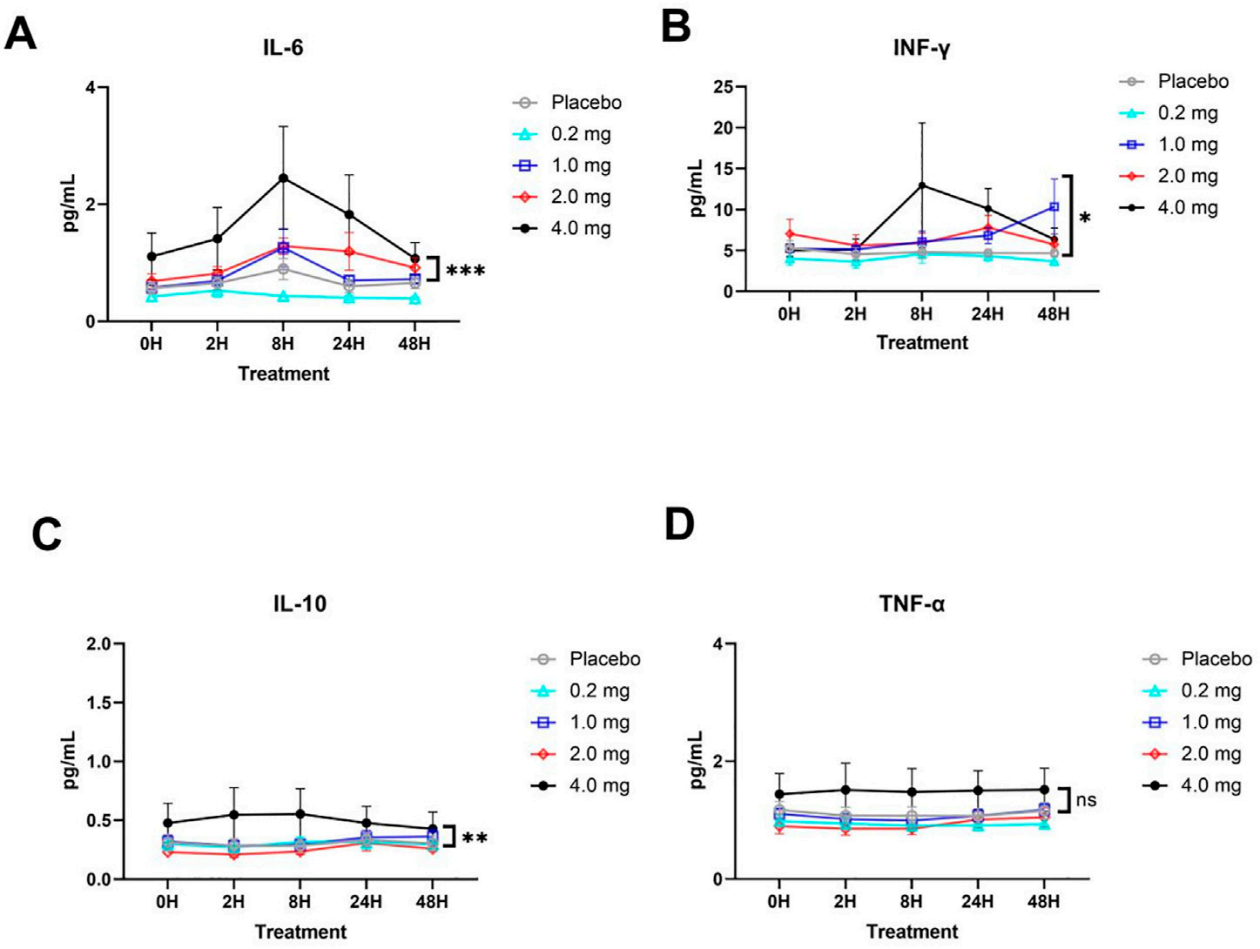
Plasma cytokine responses (mean ± SD) in each group. **(A)** IL-6 cytokine. **(B)** IFN-γ cytokine. **(C)** IL-10 cytokine. **(D)** TNF-α cytokine. The significant difference analysis was evaluated using two-way ANOVA with Tukey’s multiple comparison test (*p < 0.05, **p < 0.01, *** *p* < 0.001, ns *p* > 0.05 non significant).

No significant statistical trend in cytokine responses was observed between the 0.2, 1 and 2 mg dose groups, indicating that no significant abnormal reactions were observed from the perspective of cytokines at the 0.2–2 mg inhalation dose. At the highest dose of 4 mg, IL-6, IL-10, and IFN-γ levels gradually increased after administration and returned to the pre-administration level from 8 to 48 h (Figure 3). A high dose of 4 mg induced changes in cytokines in healthy subjects, reflecting that some adverse reactions occurred at this dose level but returned to normal after 24–48 h.

## Discussion

The worldwide outbreak of COVID-19, caused by SARS-CoV-2, has resulted in a severe challenge to the public health system. Several vaccines have been developed, however, none is preventive against infection, and more therapeutic drugs for COVID-19 are needed. The recombinant TFF2-IFN protein has antiviral effects, anti-inflammatory, and mucosal tissue repair functions. In this study, we conducted a randomized, double-blind, placebo-controlled, single-dose, dose-escalation study to investigate the safety, tolerability, immunogenicity, and PK of aerosol inhalation of recombinant TFF2-IFN protein. A total of 32 subjects were enrolled in this study, all of whom completed the experimental schedule. The primary safety assessment indicated that aerosol inhalation of recombinant TFF2-IFN had an acceptable safety profile and was generally well tolerated in healthy volunteers when administered by inhalation at doses up to 4 mg. Drug tolerability was acceptable, and maximum tolerated dose (MTD) was not observed.

All AEs were mild, transient, and resolved during the follow-up period, such as abnormal laboratory results, no symptomatic manifestations, and no need for medical intervention. AEs may related to the TFF2-IFN protein occurred three times in total, including increased Fibriogen (16.7% in the TFF2-IFN group and 0% in the placebo group), one neutrophil increase (16.7% in the nebulizer group and 0% in the placebo group), which may have resulted from the biological responsiveness of neutrophils after the application of type I IFNs (Kasimir et al., 1991; Garcia-Del-Barco et al., 2021), and fever (16.7% in the TFF2-IFN group and 0% in the placebo group), which occurred in the highest dose group (4 mg). The temperature suddenly dropped on the day of admission and administration, and the air conditioning in the room was not sufficient, which may lead to a human stress reaction. This may also cause AE, however, drug-related adverse reactions cannot be excluded, so they are considered possibly related. Nasopharyngitis (16.7% in the TFF2-IFN group and 0% in the placebo group), which was local effects, not systemically derived. The protective or detrimental effects of IFNs on patients with COVID-19 remain controversial (Zanoni, 2021). Finally, there was no withdrawal from the study due to AEs, SUSARs, or death. The safety profile of subjects receiving TFF2-IFN was similar to that of placebo-treated subjects. These data imply that TFF2-IFN is likely safe when applied by nebulization within our dosage scale.

The mean C_max_ and AUC_0–48h_ were lower for all groups, and the timing of entering the blood was observed 6–8 h post-administration at pg/ml level for C_max_ and ng/ml for AUC_0–48h_. The TFF2-IFN protein was delivered using inhaled administration, with most of the protein remaining in the lung and starting to be absorbed to the blood after 6–8 h.The local retention time is likely to increased, wiht descrease of the lower systemic exposure, which may help enhance the safety and efficacy of the TFF2-IFN protein. Biodistribution and metabolic pathway of interferon-α1b administered by aerosol inhalation in rabbits is reported same trend to absorb the interfeon-α1b (LIU Jian-feng et al., 2013).

Cytokines detection is to evaluate the level of protein-induced inflammation and keep it at an appropriate level without obviously inducing high level, and thus causing cause damage to the body. The same trend of cytokines in per group was observed and no significant statistical trend was observed in the 0.2, 1, and 2 mg dose groups, especially in the group with the highest dose (4 mg), in which IL-6, IL-10, and IFN-γ gradually increased after administration, recovering to the pre-administration level from 8 to 24 h. This implies a lower systemic exposure following nebulization of the recombinant TFF2-IFN protein, which could be translated into a lower possibility of systemic side effects in the safety profile.

The design of recombinant TFF2 and type I IFN as a protein is to simultaneously satisfy antiviral activity, alleviate the inflammatory response, and improve respiratory mucosal reconstruction. IFN-I can directly suppress viral replication (Hegde et al., 2003; Hachim et al., 2020; Hadjadj et al., 2020) and upregulate immune responses by activating antigen-presenting cells (APCs) such as dendritic cells (DCs) and inducing the expression of MHC II and costimulatory molecules in APCs, provoking natural killer and T cell proliferation and differentiation with enhanced IFN-γ secretion (Tough et al., 1996; Sun et al., 1998; Kolumam et al., 2005). In this study, the level of IFN-γ of the 4 mg group culminated at 8 h and then gradually returned to normal up to 48 h, which reflects the upregulation of immune responses by IFN-I. Interestingly, there was no increase in inflammatory responses, such as TNF-α, which is usually observed during the activation of the STAT1 pathway through the engagement of the IFN-I receptor (Muller et al., 1993; Silvennoinen et al., 1993; Darnell et al., 1994; Platanias, 2005; Gambin et al., 2013), suggesting that the recombinant TFF2-IFN protein plays a bifunctional role *in vivo*, upregulating ISGs but keeping inflammatory responses in check. In this regard, TFF2-IFN is likely to not only have an effect in the early stage of infection, but also be applicable in the late stage of infection, and further exploration will be worthwhile in the clinic in the future. Overall, the recombinant dual-functional IFN-TFF2 protein may not only have a good safety profile, but may also be effective in the treatment of respiratory viral infections, such as COVID-19. Importantly, this protein will be applicable in both the early and late stages of viral infection due to its capacity to elicit inflammatory responses.

As TFF2 is naturally expressed in mucosal tissues, we hypothesized that TFF2 is mainly functional in mucosal tissues. Therefore, the administration of the TFF2-IFN protein is preferred by inhalation, which is also the primary pathway for respiratory viral infection. A previous report showed that inhalation could achieve a higher proportion of active substances in mucosal tissues and reduce the risk of systemic side effects (Rajan et al., 2020). Consequently, our data showed that the nebulization of TFF2-IFN resulted in the retention of respiratory tracts for up to 6–8 h and only marginal release into the blood afterward, suggesting that inhalation of TFF2-IFN is a proper delivery route to cure respiratory viral infections, including COVID-19 and influenza. It is worth further clinical tests in scaled-up cohorts with diverse ethnic populations or with SARS-CoV-2/influenza infection.

This study has many limitations, including short duration of treatment, small sample size, lack of ethnic diversity, and inclusion of only healthy volunteers.

In summary, the recombinant TFF2-IFN protein was well tolerated without severe treatment-related side effects and low systemic exposure in healthy subjects after a single aerosol inhalation dose ranging from 0.2 to 4 mg/per. The main PK parameters C_max_ and AUC_0–48h_ showed low systemic exposure, even the concentration of high dose group was only pg/ml, with the data showing potential for the treatment of SARS-CoV-2 infection. Combined with the data from previous clinical trial in COVID-19, the recommended reference dose for phase Ⅱ is 1, 2 mg/per. The phase two study and future studies will further investigate the additional data from COVID-19 patients to assess the best dose. The highest dose group (4 mg) eliciting systemic IFN-γ responses in the absence of inflammatory responses may indicate that this pathway may also exert an effect on systemic viral infection, and further dose increase is possible when respiratory viral infection spills into systemic organs. Based on the results of this clinical trial, reference doses could be administered to patients with COVID-19 for phase II.

## Data Availability

The original contributions presented in the study are included in the article/Supplementary Material, further inquiries can be directed to the corresponding authors.
